# Prehypertension and Clustering of Cardiovascular Risk Factors Among Adults in Suburban Beijing, China

**DOI:** 10.2188/jea.JE20110022

**Published:** 2011-11-05

**Authors:** Wei-Hong Zhang, Lei Zhang, Wei-Feng An, Jun-Ling Ma

**Affiliations:** 1 Department of Basic Medical Sciences, Nursing College, Zhengzhou University, Zhengzhou, China; 2 Key Laboratory of Carcinogenesis and Translational Research (Ministry of Education), Department of Epidemiology, Peking University Cancer Hospital & Institute, Beijing, China; 3 Department of Social Medicine and Health Education, School of Public Health, Peking University, Beijing, China; 4 Institute of Chronic Disease Prevention and Health Education, Henan Center for Disease Control and Prevention, Zhengzhou, China

**Keywords:** prehypertension, cardiovascular, risk factor, clustering, suburban

## Abstract

**Background:**

Prehypertension is common in China and is associated with an increased risk of cardiovascular disease. The present study estimated the current prevalence of prehypertension and its association with clustering of other modifiable cardiovascular risk factors (CRFs) among adults in suburban Beijing.

**Methods:**

A cross-sectional survey of a representative sample of 19 003 suburban adults aged 18 to 76 years was carried out in 2007. Questionnaire data and information on blood pressure, anthropometric characteristics, and laboratory measurements were collected.

**Results:**

The age-standardized prevalence of prehypertension was 35.7% (38.2% in men and 31.8% in women) among adults in suburban Beijing. The prevalence of overweight/obesity, diabetes, dyslipidemia, and physical inactivity was higher in participants with prehypertension (26.7%, 4.8%, 34.3%, and 60.4%, respectively) as compared with normotensive participants (15.9%, 2.7%, 20.5%, and 29.1%, respectively), and in participants with hypertension as compared with those with prehypertension. Overall, 85.3%, 49.8%, and 17.8% of prehypertensive men had 1 or more, 2 or more, and 3 or more CRFs (overweight/obesity, diabetes, dyslipidemia, current smoking, and physical inactivity). These proportions were higher than those in normotensive men (81.5%, 45.1%, and 13.4%) and lower than those in men with hypertension (91.7%, 56.4%, 19.2%). Similar results were found when women with prehypertension were compared with women who were normotensive or hypertensive.

**Conclusions:**

A high prevalence of prehypertension and clustering of other modifiable CRFs are common among prehypertensive adults in suburban Beijing. More-effective population-based lifestyle modifications are required to prevent progression to hypertension and reduce the increasing burden of cardiovascular disease in China.

## INTRODUCTION

Prehypertension is a category between normotension and hypertension that was proposed by the Seventh Report of the Joint National Commission on Prevention, Detection, Evaluation, and Treatment of High Blood Pressure (JNC-7) in 2003^1^ and is defined as a systolic blood pressure (SBP) of 120 to 139 mm Hg or a diastolic blood pressure (DBP) of 80 to 89 mm Hg in adults aged 18 years or older. This category was suggested due to evidence indicating that overall cardiovascular risk and end-organ damage were already elevated in individuals with prehypertension, when compared with those with a BP less than 120/80 mm Hg.^2^ JNC-7 recommended close follow-up and possible intervention for individuals with prehypertension, which frequently progresses to actual clinical hypertension over several years,^3^ because, in addition to hypertension, prehypertension was found to have a strong, continuous, and graded relationship with the risk of major cardiovascular events.^1^^,^^4^

With the recent rapid economic growth and urbanization of China, the prevalences of prehypertension and hypertension have significantly increased.^5^ Three independent cross-sectional surveys of residents aged 35 to 74 years in the same rural Chinese area in 1991, 2002, and 2007 showed a prehypertension prevalence of 33.8%, 61.5%, and 54.6%, respectively.^6^ Several recent studies in China also indicate that prehypertension is common^6^^,^^7^ and that it is associated with an increased risk of cardiovascular disease.^4^ However, the association of prehypertension with clustering of other modifiable cardiovascular risk factors (CRFs) among Chinese adults has been rarely reported, particularly in more urbanized areas. The present study estimated the current prevalence of prehypertension among suburban adults in Beijing and evaluated the association of prehypertension with clustering of CRFs to provide a context for policy planners and health education programs.

## METHODS

### Study population

In 2007, a cross-sectional population survey of CRFs was carried out among adults in suburban Beijing. In brief, 3-stage stratified sampling was used to select a representative sample of the general population. In stage 1, 5 towns were randomly selected. In the second stage of sampling, 30 villages/neighborhoods were randomly selected from 76 villages/neighborhoods. In the final stage, individuals were randomly chosen from the selected townships/neighborhoods.^8^

Approximately 2 531 000 suburban residents lived in Beijing in 2007, 225 416 lived in the 5 towns we selected, and 98 512 lived in the 30 villages/neighborhoods we selected. A total of 20 655 people aged 18 to 76 years were randomly selected from the 30 primary sampling units (villages or neighborhoods) and invited to participate in the study. Of these, 968 declined to participate, 514 did not complete the study, and 180 had incomplete information. Therefore, 19 003 people (7148 men and 11 855 women) completed the survey and examinations and were eligible to be included in the analysis. The overall response rate was 92.0%. The age and sex distribution of the sample was similar to that of the total population of Beijing.

Institutional review boards or ethics committees at all participating institutes approved the study protocol. Written informed consent was obtained from each participant before data collection. Participants with untreated conditions identified during the examination were referred to a primary health care provider.

### Data collection

Data collection was conducted in examination centers at local health stations or in community clinics in the participant’s residential area. During the study visit, trained research staff administered a standardized questionnaire to obtain each subject’s demographic information, medical history, and family history, and to ascertain physical activity, alcohol use, dietary habits, and tobacco use.

### BP and anthropometric measurements

Three consecutive measurements of BP (first and fifth Korotkoff sounds) were taken on the right arm using a calibrated mercury sphygmomanometer after the participant had been resting in a seated position for at least 5 minutes; the average of these 3 measurements was used in the analysis. The room temperature was maintained at 22°C to minimize the effects of temperature and weather. Participants were advised to avoid cigarette smoking, alcohol, caffeinated beverages, and exercise for at least 30 minutes before the measurement. One of 4 cuff sizes (pediatric, regular adult, large adult, or thigh) was chosen on the basis of the participant’s arm circumference.^9^ Quality control measures for BP included repeated measures, supervisor observation of data collection, and a sphygmomanometer maintenance program. Body weight and height were measured twice during the interview. Weight was measured in light indoor clothing without shoes on electronic scales placed on a firm, level surface to the nearest 0.1 kg. Height was measured without shoes with a wall-mounted stadiometer to the nearest 0.1 cm.

### Laboratory measurements

Venous blood samples were obtained after an overnight fast and analyzed for serum lipids and plasma glucose. Participants who had not completed an overnight fast were asked to visit centers later, when their duration of fasting was longer than 10 hours. Blood samples were centrifuged at 3000 rpm for 30 minutes at 4°C, and plasma was stored at −70°C until laboratory assay. Plasma glucose and lipid levels were measured using a modified hexokinase enzymatic method (Hitachi automatic clinical analyzer, model 7060, Japan). Concentrations of total cholesterol (TC), high-density lipoprotein (HDL) cholesterol, and triglycerides (TG) were analyzed enzymatically with commercially available reagents.^10^ Lipid measurements were standardized according to the criteria of the Centers for Disease Control and Prevention–National Heart, Lung, and Blood Institute Lipid Standardization Program.^11^ For participants with a triglyceride level less than 4.5 mmol/L, low-density lipoprotein (LDL) cholesterol levels were calculated by using the Friedewald equation (LDL cholesterol = TC − HDL cholesterol − TG/5).^12^

### Criteria for data interpretation

Body mass index (BMI) was calculated as body weight (in kilograms) divided by height (in meters) squared. Overweight/obesity was defined as a BMI of 25 kg/m^2^ or higher.^13^

Diabetes was defined as a fasting plasma glucose (FPG) level of 7.0 mmol/L or higher or self-reported current treatment with antidiabetes medication (insulin or oral hypoglycemic agents).^14^

BP was classified according to JNC7^1^ recommendations. Normal BP was defined as an SBP less than 120 mm Hg and a DBP less than 80 mm Hg without use of antihypertensive medication; prehypertension as an SBP of 120 to 139 mm Hg or a DBP of 80 to 89 mm Hg without use of antihypertensive medication; and hypertension as an SBP of 140 mm Hg or higher, a DBP of 90 mm Hg or higher, or use of antihypertensive medication in the previous 2 weeks.

Dyslipidemia was defined as self-reported current treatment with cholesterol-lowering medication or having 1 or more of the following: a TC level of 5.2 mmol/L or higher, a TG level of 1.7 mmol/L or higher, an HDL cholesterol level less than 1.0 mmol/L, or an LDL cholesterol level of 3.4 mmol/L or higher.^11^

Participants who answered affirmatively to the question, “Do you smoke cigarettes now?”, and had smoked for 6 months or longer during their lifetime were classified as current smokers.^15^

A participant was classified as physically active if they took part in an exercise activity for at least 30 minutes an occasion at least 3 times per week; all other participants were classified as physically inactive.

### Statistical analysis

Data were analyzed using SAS (version 9.2, 2005, SAS Institute Inc, Cary, NC, USA), allowing for the complex survey design used for calculating the estimates. Weights were calculated using data from the 2000 China Population Census. Descriptive statistics were computed for all variables, including means for continuous variables, standard deviation (SD) of the mean, frequencies, and standard errors (SEs) for categorical variables. SEs were calculated using a technique appropriate to the complex survey design, which used strata to account for each town and village/street district. PROC SURVEYFREQ was used to estimate the prevalence of prehypertension and CRFs overall and within subgroups. Differences in continuous variables were tested using the *t* test and analysis of variance for independent samples, and prevalences for categorical variables were compared using the chi-square test and the chi-square test for trend for proportions. Odds ratios (ORs) and 95% confidence intervals (CIs)—adjusted for sex, age, family history of hypertension, and other potential confounders—were calculated by multivariate logistic regression using PROC SURVEYLOGISTIC to estimate the association between prehypertension and CRFs. All *P* values are 2-sided, and statistical significance was defined as *P* < 0.05.

## RESULTS

Approximately 5834, 6784, and 6385 participants were normotensive, prehypertensive, and hypertensive, respectively. As shown in Table 1, the age-standardized prevalence of normotension, prehypertension, and hypertension was 30.7% (26.1% in men and 35.9% in women), 35.7% (38.2% in men and 31.8% in women), and 33.6% (35.7% in men and 32.3% in women), respectively. The prevalence of prehypertension was higher in men than in women for the entire age range (*P* < 0.05 for all tests). The prevalence of prehypertension increased in age groups younger than 50 years and decreased in groups aged 50 years or older in both sexes (*P* for trend < 0.01 for all tests).

**Table 1.** Prevalence of normal blood pressure (BP), prehypertension, and hypertension among participants by sex and age

Groups	Normal BP	Prehypertension	Hypertension
Total^a^	30.7 (1.1)	35.7 (1.2)	33.6 (1.2)
Men			
Overall^a^	26.1 (0.9)	38.2 (1.2)	35.7 (1.5)
Age, y			
18–29	62.5 (2.1)	29.7 (0.7)	7.8 (0.5)
30–39	45.3 (1.5)	38.8 (0.9)	15.9 (0.9)
40–49	26.4 (1.4)	43.4 (1.1)	30.2 (1.2)
50–59	17.5 (1.5)	39.1 (1.6)	43.4 (1.7)
60–69	13.2 (1.4)	33.9 (1.6)	52.9 (2.2)
70–76	17.4 (1.7)	26.3 (1.1)	56.3 (2.5)
Women			
Overall^a^	35.9 (1.3)	31.8 (1.2)	32.3 (1.3)
Age, y			
18–29	71.2 (1.9)	24.9 (0.6)	3.9 (0.3)
30–39	58.9 (1.7)	32.1 (1.0)	9.0 (0.7)
40–49	33.9 (1.4)	37.2 (1.0)	28.9 (1.1)
50–59	23.4 (1.1)	33.8 (1.5)	42.8 (1.7)
60–69	16.9 (1.3)	28.0 (1.2)	55.1 (2.1)
70–76	17.3 (1.4)	23.1 (1.1)	59.6 (2.6)

Values are percentages (standard error).

^a^Age-standardized prevalence rate.

The demographic and metabolic characteristics of the study participants stratified by BP category are shown in Table 2. Individuals with hypertension were more likely to be male and older as compared with those with prehypertension (*P* < 0.01), and individuals with prehypertension were more likely to be male and older than normotensive adults (*P* < 0.01 for all tests). BMI, FPG, TC, TG, LDL cholesterol, and the prevalence of overweight/obesity, diabetes, dyslipidemia, and physical inactivity were all significantly higher in hypertensive subjects than in prehypertensive subjects, and in prehypertensive subjects as compared with normotensive subjects (*P* < 0.05 for all tests). Conversely, HDL cholesterol was significantly lower in hypertensive subjects than in prehypertensive subjects, and in prehypertensive subjects as compared with normotensive subjects (*P* < 0.05). Smoking status did not significantly differ among the 3 groups (*P* > 0.05).

**Table 2.** Characteristics of participants stratified by BP

Characteristics	Normal BP	Prehypertension	Hypertension	*P* value
Age (years)	45.9 ± 5.8	50.7 ± 6.4	55.2 ± 6.9	<0.01
BMI (kg/m^2^)	21.8 ± 2.9	24.2 ± 3.3	26.3 ± 3.1	<0.01
FPG (mmol/L)	5.50 ± 1.01	5.66 ± 1.05	5.70 ± 1.02	0.02
TC (mmol/L)	4.63 ± 1.09	4.81 ± 1.10	4.91 ± 1.05	<0.01
TG (mmol/L)	1.52 ± 0.52	1.68 ± 0.54	1.77 ± 0.57	<0.01
HDL-C (mmol/L)	1.39 ± 0.34	1.31 ± 0.33	1.26 ± 0.31	<0.01
LDL-C (mmol/L)	2.74 ± 0.80	2.83 ± 0.78	2.89 ± 0.82	0.03
Male	41.9 (1.1)	53.7 (1.3)	51.3 (1.5)	<0.01
Overweight/obesity	15.9 (0.2)	26.7 (0.5)	35.9 (0.8)	<0.01
Diabetes	2.7 (0.1)	4.8 (0.1)	6.9 (0.2)	<0.01
Dyslipidemia	20.5 (0.9)	34.3 (0.7)	51.4 (1.4)	<0.01
Current smoking	36.7 (1.1)	35.9 (1.2)	36.2 (1.2)	0.56
Physical inactivity	29.1 (1.1)	60.4 (1.3)	72.1 (1.1)	<0.01

Values are means ± standard deviation or percentages (standard error).

BP: blood pressure, BMI: body mass index, SBP: systolic blood pressure, DBP: diastolic blood pressure, FPG: fasting plasma glucose, TC: total cholesterol, TG: triglycerides, HDL-C: high-density lipoprotein cholesterol, LDL-C: low-density lipoprotein cholesterol.

As shown in the Figure, 25.2% (18.5% of men and 30.9% of women) of normotensive adults, 20.4% (14.7% of men and 25.8% of women) of prehypertensive adults, and 14.9% (8.3% of men and 19.5% of women) of hypertensive adults had no major modifiable CRFs. In contrast, 37.4%, 27.3%, and 10.1% of normotensive participants, 34.7%, 30.5%, and 14.4% of prehypertensive participants, and 34.4%, 34.8%, and 15.9% of hypertensive participants had 1, 2, and 3 or more CRFs, respectively. Normotensive adults were more likely to have 0 CRFs and 1 CRF in both sexes than were prehypertensive adults (*P* < 0.05 for all tests), as was the case for prehypertensive adults as compared with hypertensive adults (*P* < 0.05 for all tests). Participants with prehypertension were more likely than normotensive participants to have 2 CRFs and 3 or more CRFs (*P* < 0.05 for all tests), as was the case for hypertensive adults as compared with prehypertensive adults (*P* < 0.05 for all tests).

**Figure.**
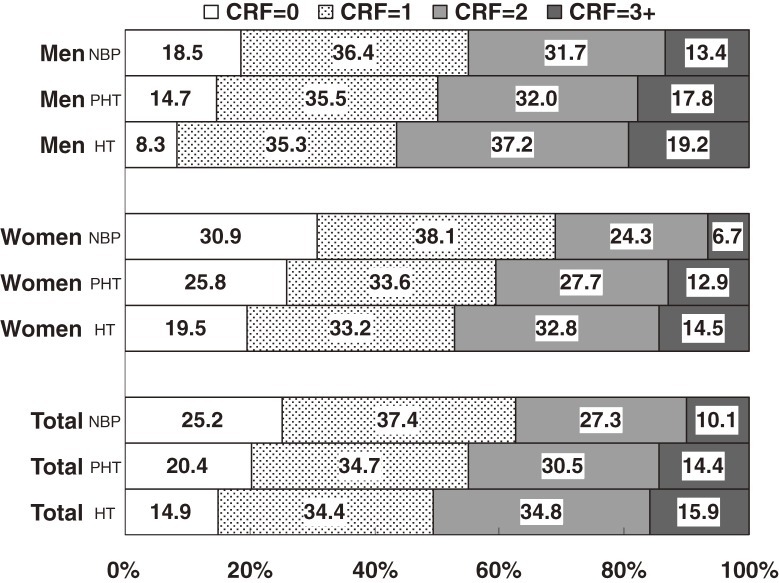
Age-standardized prevalence of CRFs among normotensive, prehypertensive, and hypertensive adults in suburban Beijing (NBP: normal blood pressure, PHT: prehypertension, HT: hypertension, CRFs: cardiovascular risk factors, ie, overweight/obesity, diabetes, dyslipidemia, current smoking, and physical inactivity).

The age-standardized prevalence rates of 1 or more, 2 or more, and 3 or more major CRFs among participants stratified by BP category are shown in Table 3. The prevalence of 1 or more, 2 or more, and 3 or more CRFs was higher in prehypertensive men (85.3%, 49.8% and 17.8%, respectively) than in normotensive men (81.5%, 45.1%, 13.4%; *P* < 0.01 for all tests), and in hypertensive men as compared with prehypertensive men (*P* < 0.01 for all tests). The prevalence of 1 or more, 2 or more, and 3 or more CRFs was also higher in prehypertensive women (74.2%, 40.6% and 12.9%, respectively) than in normotensive women (69.1%, 31.0%, 6.7%; *P* < 0.01 for all tests), and in hypertensive women as compared with prehypertensive women (*P* < 0.01 for all tests). The prevalence of 1 or more, 2 or more, and 3 or more CRFs also increased in groups younger than 60 years and decreased in groups aged 60 years or older in both sexes and all BP categories (*P* for trend < 0.01 for all tests).

**Table 3.** Prevalence of ≥1, ≥2, and ≥3 CRFs stratified by BP category in Beijing

CRFs	Men				Women			
	
	NBP	PHT	HT	*P* value	NBP	PHT	HT	*P* value
≥1 CRFs								
Overall^a^	81.5 (1.1)	85.3 (1.3)	91.7 (1.3)	<0.01	69.1 (1.0)	74.2 (1.3)	80.5 (1.2)	<0.01
Age, y								
18–29	60.6 (0.7)	64.5 (0.7)	72.4 (1.0)	<0.01	50.9 (0.6)	56.1 (0.9)	60.5 (0.8)	<0.01
30–39	70.1 (0.9)	73.0 (0.7)	82.6 (1.3)	<0.01	58.7 (0.8)	66.2 (1.0)	69.1 (0.9)	<0.01
40–49	85.0 (1.1)	89.3 (1.3)	96.0 (1.9)	<0.01	72.5 (1.1)	79.0 (1.4)	84.0 (1.2)	<0.01
50–59	89.4 (1.4)	92.8 (1.5)	99.8 (2.0)	<0.01	77.4 (1.2)	85.0 (1.5)	88.7 (1.4)	<0.01
60–69	87.3 (1.2)	90.7 (1.2)	96.2 (1.9)	<0.01	75.3 (1.2)	81.5 (1.5)	85.7 (1.2)	<0.01
70–76	79.9 (1.2)	83.5 (1.4)	87.6 (1.8)	<0.01	69.9 (1.4)	74.2 (1.7)	79.3 (1.3)	<0.01
≥2 CRFs								
Overall^a^	45.1 (0.7)	49.8 (0.9)	56.4 (1.0)	<0.01	31.0 (0.9)	40.6 (0.7)	47.3 (1.0)	<0.01
Age, y								
18–29	36.6 (0.5)	39.7 (0.7)	47.0 (0.7)	<0.01	23.5 (0.6)	31.8 (0.5)	37.9 (0.7)	<0.01
30–39	40.1 (0.5)	44.9 (1.0)	51.7 (0.9)	<0.01	27.0 (0.6)	36.2 (0.7)	43.1 (0.7)	<0.01
40–49	45.7 (0.7)	51.3 (1.1)	58.2 (1.0)	<0.01	32.1 (0.8)	42.3 (0.9)	50.8 (1.0)	<0.01
50–59	51.6 (0.9)	56.6 (1.3)	63.5 (1.3)	<0.01	38.2 (1.1)	48.5 (1.1)	56.1 (1.3)	<0.01
60–69	48.5 (1.0)	53.5 (1.3)	61.2 (1.4)	<0.01	35.9 (1.2)	45.7 (1.1)	51.9 (1.5)	<0.01
70–76	44.0 (1.2)	49.0 (1.2)	56.3 (1.3)	<0.01	31.1 (1.4)	31.6 (1.2)	47.4 (1.3)	<0.01
≥3 CRFs								
Overall^a^	13.4 (0.5)	17.8 (0.7)	19.2 (0.6)	<0.01	6.7 (0.4)	12.9 (0.6)	14.5 (0.6)	<0.01
Age, y								
18–29	8.9 (0.4)	13.0 (0.5)	14.5 (0.5)	<0.01	2.8 (0.3)	8.4 (0.5)	9.7 (0.4)	<0.01
30–39	10.1 (0.5)	14.7 (0.6)	16.3 (0.6)	<0.01	4.6 (0.3)	9.7 (0.6)	11.5 (0.5)	<0.01
40–49	14.7 (0.5)	18.9 (0.8)	20.1 (0.8)	<0.01	7.3 (0.5)	13.5 (0.7)	15.9 (0.7)	<0.01
50–59	17.1 (0.7)	21.9 (1.0)	24.3 (1.0)	<0.01	9.9 (0.7)	16.9 (0.9)	19.2 (0.9)	<0.01
60–69	15.2 (0.8)	20.0 (1.0)	22.6 (1.1)	<0.01	8.0 (0.7)	15.3 (0.9)	16.7 (1.0)	<0.01
70–76	13.8 (0.7)	17.5 (1.1)	19.4 (1.1)	<0.01	6.5 (0.8)	12.8 (0.8)	14.6 (1.1)	<0.01

Values are percentages (standard error).

CRFs: cardiovascular risk factors, BP: blood pressure, NBP: normal blood pressure, PHT: prehypertension, HT: hypertension, SE: standard error.

^a^Age-standardized prevalence rate.

## DISCUSSION

The present study estimated the current prevalence of prehypertension and its association with clustering of other modifiable CRFs among adults in suburban Beijing, China. The strengths of the present study include its enrollment of a large, representative sample of suburban dwellers in Beijing. In addition, a high response rate was achieved, standard protocols and instruments were used, training and certification requirements for data collection were strict, and a vigorous quality assurance program ensured that high-quality data were collected, all of which allowed for calculation of representative estimates.

Prehypertension affected about one third (35.7%) of suburban adults in Beijing, and the prevalence was higher in men than in women in all age categories, which is generally consistent with other published studies (30% in Jamaica,^16^ 31% in the United States,^17^ 31.6% in Korea,^18^ 32% in Japan,^19^ 35.8% in Taiwan,^20^ and 40% in the Ashanti region of Ghana).^21^ The results of these studies reveal that the prevalence of prehypertension is high in both developed and developing countries, and that prevalence in developing countries is rapidly approaching that in developed countries. Clearly, urgent attention should be paid to preventing prehypertension, particularly in developing countries experiencing changes in lifestyle. Furthermore, in the present study, the prevalence of prehypertension increased in groups younger than 50 years and decreased in those aged 50 years or older in both sexes, perhaps because most individuals in the older age groups had progressed to actual clinical hypertension. This phenomenon was also noted in a study of Americans.^17^

Prehypertension is often associated with other CRFs, such as obesity, diabetes mellitus, dyslipidemia, and phenotypes of metabolic syndrome.^22^ Participants with prehypertension in this study had significantly higher BMI, FPG, TG, TC, and LDL cholesterol, lower HDL cholesterol, and higher prevalences of other modifiable CRFs, including overweight/obesity, diabetes, hyperlipidemia, and physical inactivity, as compared with normotensive participants, which was consistent with several other studies.^23^^–^^25^ Individuals with prehypertension have an increased risk of developing hypertension and experience adverse cardiovascular consequences. Prehypertension frequently progresses to actual clinical hypertension and increases cardiovascular risk.^26^ On the basis of the JNC-7, prehypertension requires attention and health-promoting lifestyle modifications to prevent such progression.

Our findings also showed that participants with prehypertension were more likely to have more CRFs than participants with normal BP, which indicates that the prevalence and risk of having more CRFs increases with BP. Furthermore, we found a dose-response relationship between the number of CRFs and prehypertension. That is to say, a higher number of CRFs was associated with an increased OR for prehypertension as compared with normotensive participants. These findings suggest that prehypertension results from the accumulation of CRFs.

This research is limited by its reliance on estimates derived from a cross-sectional study, which did not allow quantification of the importance of prehypertension in the clustering of CRFs. This limits our ability to establish causal relationships between prehypertension and other modifiable CRFs. However, previous studies have demonstrated the importance of prehypertension to clustering of CRFs and the development of cardiovascular disease in Chinese adults.^4^

In conclusion, prehypertension and hypertension were highly prevalent in suburban Beijing, as were clusters of other modifiable CRFs. These findings suggest that prevention of prehypertension, hypertension, and cardiovascular disease requires not only BP control, but also management of other CRFs, and that decisions on antihypertensive treatment should be made after considering overall cardiovascular risk rather than BP values alone. More-effective population-based lifestyle modifications such as smoking cessation, improved diet, increased physical activity, and increased public awareness could be the most effective interventions for individuals with prehypertension in China, especially for those living in the most industrialized and urbanized areas.
